# Adult-Onset Recessive Cerebellar Ataxia and Severe Multisystem Disease-Associated Genes: Hypomorphic Alleles and Clinical Interpretation Pitfalls

**DOI:** 10.3390/genes17070758

**Published:** 2026-06-30

**Authors:** Lorenzo Cipriano, Roberta Petillo, Manuela Priolo, Paola D’Ambrosio

**Affiliations:** Medical and Molecular Genetics, AORN Cardarelli, 80131 Naples, Italy; lorenzo.cipriano@aocardarelli.it (L.C.); roberta.petillo@aocardarelli.it (R.P.); manuela.priolo@aocardarelli.it (M.P.)

**Keywords:** adult-onset cerebellar ataxia, autosomal recessive ataxia, late-onset ataxia, biallelic variants, hypomorphic alleles, residual function, neuronal ceroid lipofuscinosis, peroxisomal disorders, genetic variant interpretation, phenotype-driven filtering

## Abstract

Background: Adult-onset recessive cerebellar ataxias remain frequently unresolved after next-generation sequencing, partly because phenotype-driven filtering can deprioritize biallelic variants in genes historically associated with severe pediatric multisystem disease. This review examines how attenuated, hypomorphic, or residual-function genotypes may present as late-onset, cerebellar-predominant disease and aims to support clinical interpretation of apparently discordant recessive findings. Methods: We performed a narrative, pathway-based synthesis of autosomal recessive adult- or late-onset ataxia involving genes classically linked to lysosomal/neuronal ceroid lipofuscinosis and peroxisomal disorders. Reports were assessed for age at onset, cerebellar and extracerebellar chronology, variant class, zygosity, segregation/in-trans confirmation, biochemical or functional evidence, and implications for clinician–laboratory interpretation. Results: Published reports indicate that selected NCL-related and peroxisomal genes may be clinically relevant in adult-onset cerebellar ataxia, especially when accompanied by retinal disease, hearing loss, neuropathy, cognitive or psychiatric features, epilepsy/myoclonus, movement-disorder signs, or subtle biochemical abnormalities. Apparent phenotype–gene discordance may be compatible with residual function, allelic-series effects, and cerebellar selective vulnerability, although evidence is often limited to case reports or small series with variable segregation, biochemical, and functional support. We propose a workflow based on structured phenotype transfer, in-trans confirmation, targeted re-phenotyping, ACMG/AMP-informed evidence weighting, and avoidance of label-based exclusion from “ataxia gene” lists. Conclusions: Adult-onset recessive ataxia should not be interpreted only through canonical pediatric disease labels. A pathway-aware, phenotype-complete approach may reduce avoidable VUS retention and improve recognition of attenuated genetic presentations.

## 1. Introduction

Adult- and late-onset cerebellar ataxias remain a frequent diagnostic challenge. Although next-generation sequencing (NGS), including targeted panels, clinical exome sequencing (CES), and whole-exome sequencing (WES), has improved molecular diagnosis, many patients with apparently hereditary ataxia remain unresolved. Across adult-onset ataxia cohorts, NGS diagnostic yield typically ranges from approximately 25% to 45%, indicating that the residual gap reflects not only technical limitations but also difficulties in variant interpretation and genotype–phenotype attribution [1,2].

**Figure 1:**
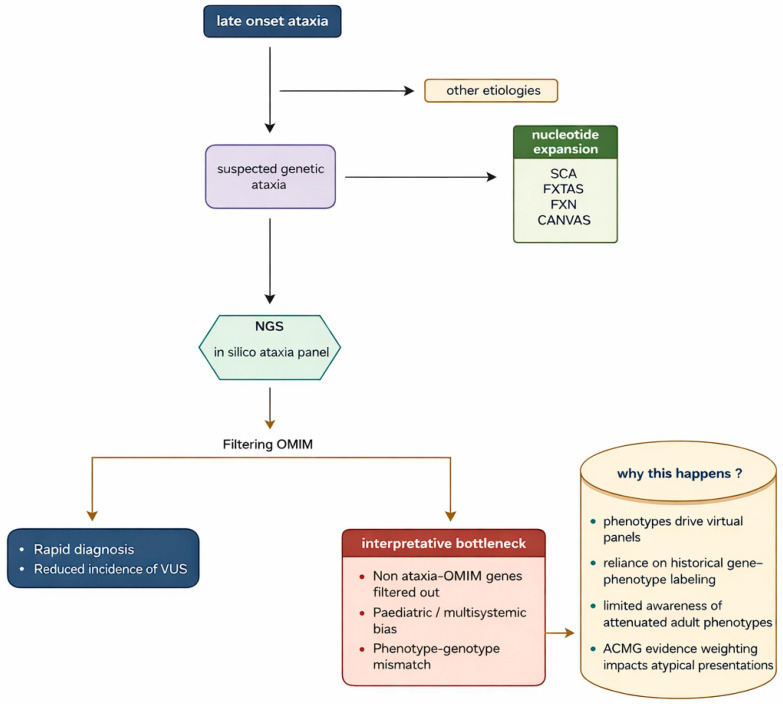
Phenotype-driven filtering and the interpretative bottleneck in late-onset ataxia. Schematic diagnostic pathway from suspected genetic ataxia through repeat-expansion testing and NGS-based analysis. Phenotype-driven virtual panels and curated-resource filtering facilitate rapid diagnoses but can exclude biallelic candidates in genes not historically labelled as ataxia-associated, contributing to VUS retention and missed attribution in attenuated adult presentations.

In routine practice, NGS interpretation is a multistep process of variant filtering, annotation, and prioritization requiring interaction among molecular biologists, bioinformaticians, and clinical geneticists (Figure 1). Because WES and large panels may identify thousands of rare variants, interpretation relies on triage strategies that reduce the search space, including targeted or virtual gene panels, phenotype-driven filters, and curated gene–disease resources [3,4]. These strategies are necessary, but they can create a predictable failure mode: variants are prioritized when they occur in genes already recognized as causative for the observed phenotype, whereas genes historically associated with different, broader, or more severe disease spectra may receive lower priority. This risk is greatest when the phenotype is incomplete, extracerebellar clues are subtle or not systematically captured, and variant-level evidence is not immediately decisive. Consequently, the main bottleneck in adult-onset ataxia diagnostics is often interpretative rather than technical, with potentially relevant biallelic variants being filtered out, retained as variants of uncertain significance (VUS), or dismissed because of perceived phenotype–gene discordance [5].

This issue is especially relevant for genes classically associated with severe early-onset multisystem disorders. In adults with isolated or predominant cerebellar ataxia, the absence of a “textbook” pediatric phenotype—such as infantile neurodegeneration, profound cognitive impairment, severe epilepsy, retinal blindness, or overt systemic involvement—may reduce confidence in causal attribution, even when recessive inheritance is plausible and biallelic configuration is confirmed or strongly suspected to be in trans [5]. Curated resources such as OMIM are indispensable for diagnostic workflows, but they necessarily reflect historical phenotypic descriptions and the timing of literature curation. Attenuated, adult-onset, or organ-restricted phenotypic expansions may therefore be underrepresented or may lag behind emerging evidence, producing a curation-dependent gene-label bias in variant triage [6].

The clinical consequence is that patients may remain labeled as idiopathic or sporadic despite plausible genetic findings, with prolonged diagnostic odyssey, limited genetic counseling, and delayed surveillance for associated features that may emerge over time. The problem is not simply the absence of genetic data, but the difficulty of recognizing non-canonical, attenuated presentations of established genetic disorders within frameworks shaped by canonical disease labels, particularly because gene–disease validity, penetrance, expressivity, and phenotypic boundaries may evolve as additional clinical, molecular, and reanalysis-based evidence accumulates [7,8,9,10,11].

A biological rationale for these adult presentations is provided by genotype–phenotype correlations, allelic series, and residual function. In selected recessive disorders, specific allelic combinations may preserve partial gene or protein function, allowing normal early development but failing to maintain long-term neuronal homeostasis. Hypomorphic variants, allelic series, and partial loss-of-function mechanisms can therefore produce later-onset, milder, or cerebellar-predominant phenotypes rather than the classical severe pediatric syndrome [12,13]. This framework is particularly relevant to lysosomal and peroxisomal pathways, where chronic partial dysfunction may affect vulnerable neuronal populations over decades.

This review focuses on autosomal recessive adult- or late-onset cerebellar ataxias involving genes not classically considered primary ataxia genes, with emphasis on lysosomal/neuronal ceroid lipofuscinosis (NCL)-related genes and peroxisomal biogenesis or metabolism genes [14]. The aim is not to expand ataxia gene lists indiscriminately, but to clarify when apparent phenotype–gene discordance is biologically plausible and clinically actionable. We synthesize case-level evidence in these pathway groups, with attention to extracerebellar features, chronology, variant-level plausibility, and residual-function mechanisms, and propose a clinician–laboratory workflow based on targeted re-phenotyping, confirmation of recessive configuration, and cautious integration of residual-function biology into variant interpretation [3,4,5,6,12,15].

## 2. Biological Rationale for Adult-Onset, Cerebellar-Predominant Recessive Phenotypes

**Figure 2:**
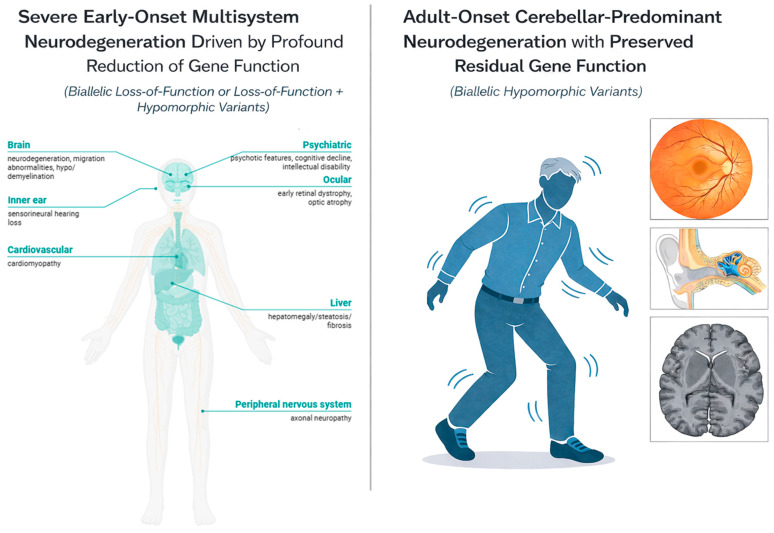
Residual-function model for attenuated adult presentations. Conceptual comparison between profound reduction in gene function (typically associated with severe early-onset multisystem disease) and preserved residual function (often compatible with adult-onset, cerebellar-predominant neurodegeneration due to biallelic hypomorphic genotypes), with extracerebellar features that may be mild or delayed.

Adult/late-onset autosomal recessive ataxia in genes historically associated with severe pediatric syndromes is often biologically plausible rather than “discordant.” Two convergent concepts—allelic series with residual function and cerebellar selective vulnerability—help explain cerebellar-predominant adult presentations and justify keeping “non-canonical” recessive genes on the table when biallelic variants are found. A residual-function framework linking allele severity to clinical presentation is illustrated in Figure 2.

### 2.1. Literature Search and Article Selection

A PubMed search was performed for publications indexed from 1 January 1900 to 1 May 2026. The search strategy combined phenotype-, onset-, inheritance-, pathway-, and gene-based terms, including “ataxia,” “cerebellar ataxia,” “spinocerebellar ataxia,” “adult-onset,” “late-onset,” “delayed onset,” “attenuated,” “hypomorphic,” “residual function,” “autosomal recessive,” “biallelic,” “homozygous,” “compound heterozygous,” “neuronal ceroid lipofuscinosis,” “NCL,” “CLN,” “lysosomal,” and “peroxisomal.” Additional gene-specific searches were performed for NCL-related and peroxisomal genes relevant to adult or late-onset cerebellar presentations, including PPT1, TPP1, CLN3, CLN5, CLN6, MFSD8/CLN7, CLN8, CTSD/CLN10, GRN/CLN11, ATP13A2/CLN12, KCTD7/CLN14, PEX7, PEX10, PEX11B, HSD17B4, PHYH, and additional peroxisomal genes identified through cross-references from selected articles.

Adult-onset was defined as onset of cerebellar ataxia, or of the first disease-relevant neurological manifestation, at ≥18 years. Late-onset was used for presentations occurring later than the classical pediatric disease window of the corresponding gene or disorder, including attenuated phenotypes in which cerebellar ataxia emerged after longstanding extracerebellar manifestations. Articles were selected when they reported human patients with adult- or late-onset cerebellar ataxia, or clinically relevant cerebellar involvement within an attenuated presentation, together with biallelic variants in genes historically associated with severe pediatric multisystem disorders, particularly NCL-related or peroxisomal genes. Selection required sufficient clinical and molecular detail for genotype–phenotype interpretation, including the affected gene, variant nomenclature, zygosity, and, when available, segregation/in-trans confirmation, biochemical abnormalities, genotype–phenotype correlations, or functional evidence. Articles were excluded when they did not meet these criteria, described only classical severe pediatric presentations without relevance to adult or attenuated disease, or reported ataxia as incidental or insufficiently characterized. Duplicate or overlapping reports were considered only when they provided additional clinical follow-up, segregation data, biochemical findings, or functional validation.

For each selected report, relevant clinical and molecular information was recorded when available, including age at onset, presenting neurological feature, disease chronology, cerebellar signs, ocular involvement, neuropathy, hearing loss, cognitive or psychiatric manifestations, seizures or myoclonus, biochemical abnormalities, neuroimaging, gene, variant class, zygosity, segregation/in-trans confirmation, functional evidence, and reported genotype–phenotype interpretation.

### 2.2. Allelic Series and Residual Function: Severity as a Quantitative Output

**Figure 3:**
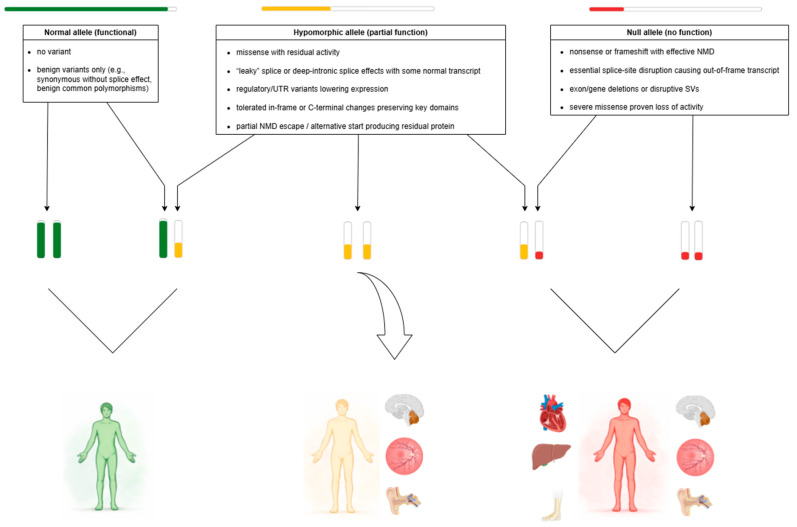
Allelic series framework linking functional allele state to phenotypic severity in autosomal recessive disease. Schematic classification of normal, hypomorphic, and null allele states with representative variant mechanisms (regulatory/UTR effects, leaky splicing, tolerated in-frame/C-terminal changes, partial NMD escape/alternative initiation versus effective loss-of-function by nonsense/frameshift with NMD, essential splice disruption, or disruptive structural variants).

In autosomal recessive disease, age at onset and severity can be interpreted as a quantitative consequence of net residual function across both alleles, making hypomorphic genotypes a coherent explanation for adult-onset, organ-predominant phenotypes in genes classically associated with severe pediatric multisystem syndromes. In an inherited ataxia cohort (366 families), hypomorphic variants significantly contributed to phenotypic heterogeneity. Among them “pseudo–loss-of-function” variants, annotated as truncating but still compatible with residual function (e.g., tolerated C-terminal truncation or translation reinitiation), should be considered as a recurring mechanism compatible with attenuated adult presentations [12]. These findings are consistent with the new refined recommendations that suggest to apply LoF evidence (PVS1) (and consequently “truncating = null = severe/early” causative variant), only after transcript- and mechanism-aware assessment (including NMD considerations and alternative transcripts) because premature termination codons do not uniformly generate true null alleles [16]. Mechanistically, residual function can be preserved through multiple routes that map to the operational categories in Figure 3: missense changes retaining partial activity; “leaky” splice or deep-intronic splice-altering variants that still allow a fraction of correctly spliced transcript [17]; regulatory/UTR variants reducing expression without abolishing coding potential [18]; tolerated in-frame or C-terminal changes sparing critical domains [12]; and context-dependent “truncating” outcomes in which residual protein is produced via partial NMD escape or alternative initiation/translation reinitiation (both mechanisms demonstrated in human disease for NEMO and IL1RN) [19,20]. Taken together, all the mechanisms exemplified in Figure 3 should be considered during the variant interpretation process in the light of possible genotype-phenotype correlation. In the context of adult onset cerebellar-predominant ataxias, the identification of biallelic variants in “pediatric” multisystem genes, should prompt interpretation and possible functional validation of a specific allele combination eventually compatible with residual function rather than merely considering if that gene is historically ataxia-centred [12,16].

### 2.3. Cerebellar Selective Vulnerability: Why Chronic Homeostatic Stress Can Look Cerebellar-Predominant

A complementary explanation for cerebellar-predominant adult presentations is selective vulnerability within a heterogeneous cerebellum, where specific Purkinje-cell subpopulations and circuits fail under chronic stress despite broad gene expression. Region- and cell-type–restricted pathology has been demonstrated in genetic ataxia models: in SCA1 knock-in mice, Purkinje cell and glial pathology emerges earlier and more severely in defined cerebellar regions, consistent with intracerebellar heterogeneity shaping disease expression [21]. Neuropathological analyses across human ataxias also support region-specific differences in Purkinje cell involvement, reinforcing selective vulnerability as a recurring biological feature rather than an exception [22]. Diverse genetic insults converge on Purkinje dysfunction as a common endpoint, and physiological studies show that Purkinje firing abnormalities can precede overt degeneration and contribute directly to motor impairment, providing a plausible relation between chronic cellular stress, circuit failure, and the clinical ataxia phenotype [23,24]. Autophagy–lysosome homeostasis offers a particularly coherent substrate for this vulnerability: disruption of core autophagy genes causes cell-autonomous Purkinje neurodegeneration in vivo [25], ATG5-dependent autophagy preserves Purkinje survival and cerebellar function [26], and pathogenic ATG5 variation in humans causes a syndromic ataxia phenotype, anchoring this axis as clinically relevant [27]. Together, these lines of evidence support a clinically relevant inference for this review: chronic, partial impairment of homeostatic pathways—particularly those intersecting autophagy–lysosome function—can preferentially compromise cerebellar circuits, providing a biologically grounded basis for adult, cerebellar-predominant phenotypes in disorders otherwise framed as broader multisystem disease. Cellular stress responses may also intersect with DNA-damage checkpoint pathways and p21–ASK1 stress-checkpoint signalling, which regulate the balance between arrest, senescence, apoptosis, and survival under persistent stress [28,29].

## 3. Pathway-Based Exemplar Selection and Analytic Framework

Adult-onset autosomal recessive ataxia is typically analysed through phenotype-driven triage (virtual panels, curated gene lists) followed by ACMG/AMP classification in which phenotype specificity and genotype–phenotype acknowledged association directly influence variant interpretation and their reportability [5]. This workflow is necessary for throughput, but it creates a predictable vulnerability: biallelic candidates in genes historically framed as severe paediatric multisystem disorders are less likely to be prioritised when the adult presentation is cerebellar-predominant and extracerebellar features are not systematically captured or transferred to the laboratory [3,4].

We will focus on two gene groups in which adult or late-onset cerebellar presentations are documented and where the phenotype commonly includes “low-salience” extracerebellar cues (ocular disease, seizures/cognitive change, neuropathy, hearing loss, mild biochemical signals) in order to provide a pathway-based exemplar strategy that could eventually be generalized to other disorders. This selection is grounded in the established genetic heterogeneity and phenotypic breadth of both families: NCL disorders generally span childhood and adult forms with marked variability in both prominence and timing of motor, retinal, epileptic, and cognitive features [30,31,32]. Similarly, peroxisomal disorders include overlapping biogenesis and single-enzyme/protein defects for which integrated biochemical–genetic interpretation is explicitly recommended in technical standards and reviews [33].

Analytically, each gene section is structured around elements that are directly usable in interpretation and reporting: (i) historical phenotype framing in curated resources, (ii) adult/late-onset case-level evidence with biallelic genotypes, (iii) sequence of feature occurrence and timing of symptom onset (ataxia-first vs. retina-first vs. broader onset), (iv) objective extracerebellar findings and their timing, and (v) variant-level context relevant to attenuated mechanisms and residual function.

### 3.1. Lysosomal/NCL-Related Genes: Adult Cerebellar Presentations Within the NCL Spectrum

Neuronal ceroid lipofuscinoses are genetically heterogeneous lysosomal disorders which are classically characterized by variable combinations of cognitive decline, epilepsy, visual loss and motor deterioration [32] with broad age-at-onset distributions, including established adult forms and attenuated phenotypes in which retinal, epileptic, cognitive/psychiatric, and motor features vary in occurrence, severity and onset [30,31,32]. In adult neurology, this variability is clinically relevant because cerebellar ataxia may represent the first occurring sign or be the predominant one, while the other “canonical” paediatric features may be absent, subtle and unrecognized or even emerge later.

To date, at least fourteen different NCL forms have been described inherited as both autosomal dominant and recessive traits [32] CLN4/DNAJC5 is an autosomal dominant cause of adult-onset NCL and is therefore outside the recessive focus of this review. Accordingly, this section and Table 1 include only recessive NCL-related genes with reported adult or attenuated cerebellar presentations.

Here we provide a list of recently identified adult-onset forms (Table 1) presenting with mild features [40].

CLN1/PPT1: Adult-onset CLN1/PPT1 disease provides a functionally supported example of residual-function biology within the NCL spectrum: patients with palmitoyl-protein thioesterase 1 deficiency have been reported with psychiatric or cognitive onset in adulthood followed by visual decline and cerebellar involvement, with markedly reduced but residual PPT1 activity supporting an attenuated adult presentation [41,42].

CLN2/TPP1: Although classical CLN2 disease is typically late-infantile, biallelic TPP1 variants have also been associated with autosomal recessive spinocerebellar ataxia type 7, a slowly progressive ataxic phenotype distinct from classical rapidly progressive CLN2 disease; this supports the broader principle that NCL-related genes may enter ataxia workflows through attenuated non-classical presentations [36].

CLN5: Adult-onset autosomal recessive cerebellar ataxia associated with biallelic CLN5 variation was described in two siblings with onset after age 50; ocular symptoms preceded neurological manifestations, and the late attenuated phenotype was considered compatible with a hypomorphic mechanism [34].

CLN6: In a cohort of 20 cases from 13 families, CLN6-related Kufs disease showed mean onset in early adulthood with a wide range including midlife, typically presenting with progressive myoclonus epilepsy; motor disturbance, including ataxia, is part of the clinical spectrum and is relevant when adult cases are triaged through an ataxia-centric lens [35].

MFSD8 (CLN7): Adult-onset ataxia with maculopathy/retinal dystrophy has been documented with biallelic MFSD8 variants, including both ataxia-first and vision-first trajectories across adult cases [37]. In parallel, non-syndromic adult-onset macular dystrophy has also been reported with MFSD8 variants, supporting a broad adult phenotypic spectrum in which retinal involvement may occur independently of overt neurological disease [38]. A five-patient case series focused on adult-onset cerebellar ataxia in MFSD8-related disease further supports MFSD8 as a recurrent adult ataxia candidate within the NCL spectrum [14].

CTSD (CLN10): Biallelic CTSD variants were reported in a patient with retinitis pigmentosa and exceptionally late-onset cerebellar ataxia, illustrating long lead-times between retinal and cerebellar phenotypes and a diagnostic entry through movement-disorders services [39].

Across these exemplars, the recurrent interpretative point is not syndromic re-labelling but chronology: adult NCL-related presentations can be cerebellar-predominant with retinal or epileptic/cognitive features that are subtle, delayed, or not volunteered without targeted enquiry [34,35,37,39].

Selected neuronal ceroid lipofuscinosis-related genes with reported adult or late-onset phenotypes relevant to cerebellar ataxia. The table contrasts the classical disease frame with adult ataxia-relevant presentations, reported variant patterns, and available functional or residual-function evidence. Abbreviations: AD, autosomal dominant; AR, autosomal recessive; CLN, ceroid lipofuscinosis neuronal; ERG, electroretinography; NCL, neuronal ceroid lipofuscinosis; SCAR7, autosomal recessive spinocerebellar ataxia type 7; VUS, variant of uncertain significance.

### 3.2. Peroxisomal Biogenesis and Peroxisomal Metabolism Genes

Peroxisomal disorders provide a second pathway-based example of adult or late-onset cerebellar ataxia occurring within disease spectra usually framed as early-onset multisystem conditions. Compared with the NCL-related examples, the available evidence is more heterogeneous and is often based on single families or small series (Table 2). Therefore, peroxisomal genes should be considered cautiously, and mainly when recessive ataxia co-occurs with ocular disease, neuropathy, hearing loss, cognitive involvement, movement-disorder features, or peroxisomal biochemical abnormalities.

The principal autosomal recessive examples relevant to this review are PEX10, PEX7, HSD17B4, and PEX11B. Biallelic PEX10 variants have been reported in autosomal recessive ataxia with cerebellar atrophy, peripheral neuropathy, posterior column involvement, and mild or atypical biochemical abnormalities, supporting consideration of peroxisomal dysfunction in selected adult or attenuated recessive ataxia presentations [43]. PEX7 variants have been described in siblings with congenital cataract/retinopathy followed decades later by progressive ataxia and cognitive impairment, illustrating the diagnostic relevance of long intervals between extracerebellar and cerebellar manifestations [44]. Biallelic HSD17B4 variants have been identified in middle-age-onset, slowly progressive spinocerebellar ataxia, often accompanied by hearing loss, with functional evidence supporting an attenuated D-bifunctional protein phenotype [45]. PEX11B pathogenic variants have more recently been associated with an expanded adult movement-disorder phenotype including ataxia, tremor, dystonia, neuropathy, hearing impairment, and mild biochemical abnormalities, although this evidence remains limited and should be interpreted cautiously [46].

Selected autosomal recessive peroxisomal genes with reported adult or late-onset phenotypes relevant to cerebellar ataxia. The table contrasts the classical disease frame with adult ataxia-relevant presentations, reported variant patterns, and available biochemical, functional, or residual-function evidence. Abbreviations: AR, autosomal recessive; DBP, D-bifunctional protein; PBD, peroxisome biogenesis disorder; PTS2, peroxisomal targeting signal 2; RCDP1, rhizomelic chondrodysplasia punctata type 1

Other autosomal recessive peroxisomal disorders provide useful diagnostic context, but should be separated from the core exemplars summarized in Table 2. Adult Refsum disease, most often related to PHYH and less frequently to PEX7, is a recognized adult peroxisomal disorder in which ataxia may occur with retinitis pigmentosa, anosmia, neuropathy, hearing loss, ichthyosis, and elevated phytanic acid. In addition, selected mild Zellweger-spectrum or single-enzyme peroxisomal disorders may include late neurological manifestations, although adult cerebellar-predominant ataxia is less consistently documented. These conditions support the broader diagnostic principle that peroxisomal disease should be considered when ataxia is accompanied by ocular, neuropathic, auditory, cognitive, movement-disorder, or biochemical clues; however, they are not treated here as core evidence for the recessive adult-onset ataxia framework.

The main interpretative point is not to expand ataxia panels indiscriminately, but to recognize a specific pattern: adult-onset recessive ataxia with low-salience extracerebellar features or peroxisomal biochemical abnormalities. In this setting, peroxisomal genes may warrant targeted re-evaluation and phenotype completion. Table 2 summarizes the principal autosomal recessive peroxisomal examples retained as core exemplars in this review [33,43,44,45,46].

## 4. Practical Phenotypic Cues and an Interpretation Workflow for Adult-Onset Ataxia in Lysosomal and Peroxisomal Gene Spectra

The following sections provide a minimal, practice-oriented protocol—phenotypic triggers, low-friction tests, and reporting steps—to support interpretation of plausible recessive findings in adult-onset ataxia [5].

### 4.1. Phenotypic Red Flags That Should Trigger Targeted Re-Phenotyping (And Prevent Premature Variant Deprioritization)

The following findings should prompt targeted re-phenotyping and re-appraisal of pathway-relevant candidates when adult-onset cerebellar ataxia is evaluated:

- Ocular disease (often underreported unless actively searched): maculopathy/retinal dystrophy, night blindness, congenital cataract or childhood ocular disease, abnormal fundus/OCT, or abnormal/absent ERG. Retina-first or long-lead trajectories are documented in both groups [14,34,37,39,44].
- Hearing loss (including subtle or late-onset): should be treated as phenotype-relevant when combined with progressive ataxia and/or neuropathy in attenuated peroxisomal/metabolic spectra [45,46].
- Peripheral neuropathy or sensory complaints: sensory complaints, reduced vibration sense, areflexia, neuropathic pain, or prior abnormal studies should trigger objective characterization; defining the neuropathy materially sharpens phenotype attribution in adult ataxia pathways [43,46,47].
- Cognitive/psychiatric features (often delayed or initially subtle): executive dysfunction, behavioural change, or progressive cognitive decline may be mild early; its presence increases coherence with adult NCL/Kufs trajectories and selected peroxisomal presentations [34,35,44].
- Family structure consistent with recessive disease: consanguinity/endogamy, multiple affected siblings, or geographic isolate should increase scrutiny of biallelic findings beyond pre-defined “ataxia gene” sets [3,5].

### 4.2. Reporting Workflow: Minimal Requirements to Avoid Losing Clinically Relevant Biallelic Findings

This review addresses a recurrent diagnostic pitfall in adult-onset ataxia: plausible biallelic variants in genes historically associated with severe pediatric multisystem disease are down-weighted when phenotype information is incomplete and phenotype–gene concordance is judged against canonical expectations [5]. The following minimum workflow elements are designed to reduce that risk in routine practice [3,4,5,6].

1. Structured phenotype transfer (neurology → laboratory): provide, at minimum, age at onset/tempo, ocular history, hearing status, neuropathy features (±NCS/EMG summary if performed), cognition/psychiatric features, MRI summary, and family structure (including consanguinity/isolate when relevant). Phenotype information directly impacts evidence weighting and reportability under ACMG/AMP standards [5].
2. Mandatory documentation of recessive configuration: for recessive attribution, confirm biallelic configuration in trans by segregation or phasing whenever feasible, and explicitly state the phasing status in the report (confirmed in trans vs. unresolved) [5].
3. Avoid label-based exclusion (“not OMIM-ataxia”): absence of an ataxia-centred label in curated resources should not be used to exclude a plausible biallelic finding; instead, it should trigger targeted completion of the objective phenotype domains highlighted above (OCT/ERG, audiology, NCS/EMG as indicated) to test genotype–phenotype coherence [3,6].
4. Closed-loop clinician–laboratory iteration before sign-out: if the laboratory identifies a plausible recessive finding in a “non-canonical” gene, targeted re-phenotyping should be requested and incorporated before final report sign-out; conversely, if clinical red flags are present, gene prioritization should not be restricted to ataxia-tagged lists [3,4,5].

**Figure 4:**
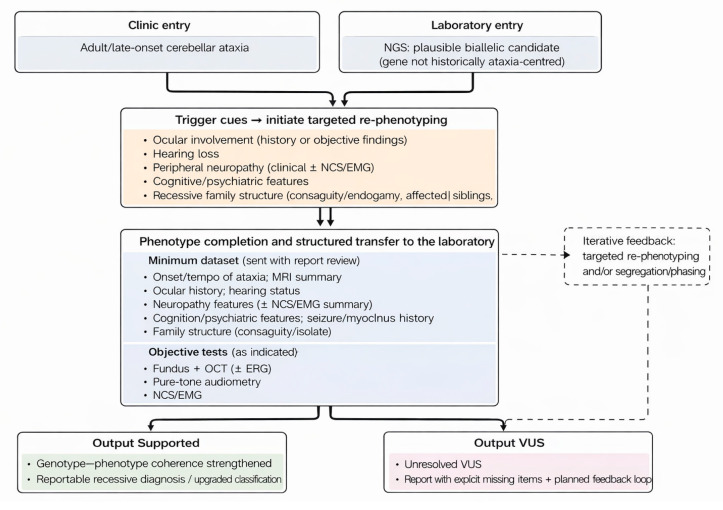
Structured clinician–laboratory feedback loop for adult-onset recessive ataxia.

To align the proposed workflow with ACMG/AMP-informed variant classification, Figure 4 summarizes the evidence categories most relevant to biallelic findings in adult-onset recessive ataxia. In this setting, classification is most influenced by confirmation of in-trans configuration, segregation, population frequency compatible with recessive disease, transcript-aware assessment of loss-of-function variants, functional or biochemical evidence, and phenotype specificity beyond isolated ataxia.

The workflow shows how a clinical presentation of adult/late-onset cerebellar ataxia or the laboratory detection of a plausible biallelic candidate in a non-canonical ataxia gene should trigger targeted re-phenotyping. Ocular involvement, hearing loss, neuropathy, cognitive/psychiatric features, and recessive family structure are used to complete the phenotype and guide objective testing. Structured transfer of these data to the laboratory can improve genotype–phenotype assessment, support a reportable recessive diagnosis or variant reclassification, and identify cases that should remain VUS pending additional clinical, segregation, or functional evidence.

## 5. Related Genes Not Yet Recognized as Adult-Onset Ataxia Genes

Although this review focuses on a core set of lysosomal/NCL-related and peroxisomal genes with published adult/late-onset cerebellar presentations, the interpretative challenge is not restricted to those exemplars. Both disease families are genetically heterogeneous and encompass broad spectra of severity and age at onset; curated syntheses and contemporary reviews explicitly document ongoing phenotypic expansion as additional alleles and cohorts are reported [30,31,32]. In parallel, large diagnostic cohorts in inherited ataxia show that hypomorphic and residual-function mechanisms are common and can shift expected onset and phenotypic emphasis, supporting the expectation that additional loci within convergent pathways may become relevant to adult cerebellar phenotypes as evidence accumulates [12].

A secondary purpose of this review is therefore to encourage a pathway-aware posture in adult-onset ataxia interpretation and reporting. When biallelic variation is identified and the phenotype includes extracerebellar cues that recur in these pathways—particularly retinal/macular involvement, epilepsy/myoclonus, neuropsychiatric/cognitive change, neuropathy, or hearing loss—candidate consideration should extend beyond historical “ataxia gene” sets to include other genes already established within NCL or peroxisomal disease frameworks, while maintaining conservative gene-level attribution when adult-specific evidence remains limited. Within the NCL spectrum, curated resources and recent reviews provide an explicit map of gene membership and phenotypic breadth and support inclusion of additional candidates such as TPP1/CLN2, CLN3, CLN8, GRN/CLN11, ATP13A2/CLN12, and KCTD7/CLN14 during re-analysis in adults with compatible multisystem cues [31,32]. For peroxisomal disease, technical standards emphasize heterogeneity across peroxisome biogenesis defects (multiple PEX genes) and single enzyme/protein defects and the need for integrated biochemical–genetic interpretation; gene–disease validity curation further underscores that clinically used peroxisomal gene sets are evidence-graded and periodically re-evaluated as new data emerge [33,48]. Together, these considerations support a structured approach to longitudinal phenotype capture and periodic re-interpretation in unresolved adult-onset ataxia, enabling incorporation of emerging gene–phenotype relationships as they become clinically substantiated.

## 6. Discussion

A substantial fraction of adult- and late-onset cerebellar ataxia remains without a definitive molecular diagnosis despite widespread use of NGS [15]. This gap is not only technical. A recurrent interpretative problem is that plausible biallelic variants in genes historically associated with severe pediatric multisystem disease may be deprioritized, retained as VUS, or dismissed when the adult presentation is cerebellar-predominant and does not fit canonical syndrome expectations [3,4,5]. Phenotype-driven triage, virtual panels, curated resources, and reporting heuristics remain necessary, but they can reduce the visibility of attenuated or overlapping presentations during variant interpretation, particularly in hereditary motor disorders where molecular diagnosis has progressively challenged historical boundaries between ataxias, spastic paraplegias, and related complex phenotypes [6,49,50,51].

The NCL-related and peroxisomal exemplars reviewed here show the same diagnostic pattern. Adult disease may present primarily as progressive ataxia, whereas retinal involvement, seizures, cognitive or psychiatric features, neuropathy, hearing loss, or biochemical abnormalities may be subtle, delayed, temporally dissociated, or not actively investigated [14,34,35,39,43,44,45,46]. Therefore, absence of a classical pediatric NCL or peroxisomal phenotype should not be used as an exclusion criterion in adult ataxia. Attribution may fail when interpretation is based on an “ataxia-only” snapshot rather than on longitudinal reconstruction and targeted assessment of extracerebellar signs.

Allelic series, residual function, and cerebellar selective vulnerability provide a plausible biological framework for these attenuated presentations. Hypomorphic genotypes can reconcile adult-onset, organ-predominant disease with genes otherwise associated with severe early-onset syndromes [12]. Chronic partial impairment of neuronal homeostatic pathways, including autophagy–lysosome function, may preferentially affect cerebellar circuits despite broader gene expression [23,24,25,26,27]. These mechanisms support biological plausibility, but they do not establish causality for every reported variant; attribution still requires convergence between biallelic configuration, variant-level evidence, phenotype coherence, and longitudinal clinical data.

The practical implication is procedural. Adult-onset recessive ataxia interpretation should combine structured deep phenotyping, explicit documentation of recessive in-trans configuration, and closed-loop clinician–laboratory reassessment before plausible biallelic findings are downgraded. High-yield domains include OCT ± ERG, audiology, and neurophysiology when neuropathy is suspected, with complete transfer of these data to the diagnostic laboratory [4,5]. This approach may reduce avoidable VUS retention without indiscriminate expansion of ataxia gene lists, particularly when variant interpretation is supported by independent clinical, biochemical, functional, or longitudinal biomarker evidence rather than by genotype alone. Conversely, the absence of phenotype-concordant objective evidence should caution against attributing pathogenic relevance to nonspecific VUS in genes with broad or incompletely defined disease spectra [5,52,53,54].

Important limitations remain. Evidence for individual adult-onset lysosomal and peroxisomal presentations is often based on case reports or small series, and segregation, phasing, biochemical confirmation, and functional validation are variably available [34,37,46]. The proposed framework should therefore be applied conservatively: pediatric gene framing should not be exclusionary when plausible biallelic variants are observed, but diagnostic attribution should remain contingent on phenotype coherence, recessive configuration, objective extracerebellar findings, explicit uncertainty, and reassessment as additional evidence becomes available. Future neurogenetic workflows may further improve this process by integrating structured phenotyping, genomic reinterpretation, biochemical or functional readouts, and artificial-intelligence-assisted prioritization within longitudinal, multimodal patient models [55,56,57,58].

## Figures and Tables

**Table 1:** NCL-related genes in adult-onset ataxia: classical disease frame, adult phenotypes, and residual-function evidence.

CLN/Gene	Classical Frame	Adult/Late-Onset Ataxia Relevance	Adult-Associated Variants	Functional/Residual-Function Evidence
**CLN1/PPT1 (AR)**	Infantile NCL; visual/psychomotor decline, seizures, ataxia	Adult onset with psychiatric/cognitive symptoms, later visual decline and cerebellar involvement	p.Arg151Ter + p.Gly108Arg; p.Arg151Ter + p.Cys45Tyr	Direct enzymatic evidence: markedly reduced but residual PPT1 activity [34,35]
**CLN2/TPP1 (AR)**	Late-infantile CLN2; seizures, regression, visual loss, motor decline	SCAR7: slowly progressive recessive spinocerebellar ataxia, distinct from classical CLN2	c.509-1G>C + c.1397T>G	Reduced TPP1 activity; residual activity supports attenuated presentation [36]
**CLN5 (AR)**	Childhood CLN5; dementia/regression, seizures, visual failure, motor decline	Adult-onset cerebellar ataxia, including onset after age 50; ocular symptoms may precede ataxia	Homozygous missense variants: p.Ser312Asn; p.Phe188Leu	Pathogenicity/trafficking data for p.Ser312Asn; hypomorphic effect compatible/proposed, not directly quantified [34]
**CLN6 (AR)**	Variant late-infantile NCL; seizures, visual loss, regression, motor decline	Adult/Kufs disease; ataxia is a prominent motor feature	Homozygous or compound heterozygous CLN6 variants; lower truncating burden than late-infantile CLN6	Clinical-genetic/pathological evidence; residual-function inferred [35]
**MFSD8/CLN7 (AR)**	Late-infantile CLN7; seizures/motor onset, regression, myoclonus, visual loss	Adult cerebellar ataxia ± maculopathy; ataxia-first, vision-first, or retina-restricted adult presentations	p.Ile312Thr; p.Glu336Gln with p.Arg482Ter or p.Arg465Gln; p.Gly52Arg + p.Glu336Gln in isolated maculopathy	Residual/dosage effect inferred; functional studies limited in adult ataxia series [14,37,38]
**CTSD/CLN10 (AR)**	Congenital/infantile-to-juvenile CLN10; visual and motor neurodegeneration	Retinitis pigmentosa followed years later by exceptionally late-onset cerebellar ataxia	p.Ala20SerfsTer25 + p.Thr355Met	Direct functional evidence: reduced but residual Cathepsin D activity, impaired processing/autophagic flux [39]

**Table 2:** Peroxisomal genes in adult-onset ataxia.

Peroxisomal Gene	Classical Frame	Adult/Late-Onset Ataxia Relevance	Adult-Associated Variants	Functional/Residual-Function Evidence
**PEX10 (AR)**	Zellweger spectrum/peroxisome biogenesis disorder; classically severe early-onset multisystem disease	Autosomal recessive ataxia with cerebellar atrophy, axonal motor neuropathy, and posterior column dysfunction	Compound heterozygous variants: c.764_765insA + c.992G>A; c.2T>C + c.790C>T	Mild/atypical biochemical abnormalities; catalase mosaicism; rescue after PEX10 cDNA transfection [43]
**PEX7 (AR)**	RCDP1/PTS2 receptor defect; classically skeletal, ocular, and developmental disease	Congenital cataract/retinopathy followed decades later by progressive ataxia and cognitive impairment	Homozygous c.35T>C/p.Leu12Pro	Elevated phytanic acid; residual-function mechanism inferred from mild course; no parental segregation available [44]
**HSD17B4/DBP (AR)**	D-bifunctional protein deficiency; classically severe infantile peroxisomal disease	Middle-age-onset slowly progressive spinocerebellar ataxia, often with hearing loss and mild sensory involvement	Homozygous c.523G>A/p.Ala175Thr	Normal mRNA with reduced DBP protein and reduced dimerized DBP; residual function proposed [45]
**PEX11B (AR)**	Peroxisome biogenesis disorder 14B; classically developmental/multisystem disease	Adult-onset ataxia with tremor/dystonia, myoclonus, neuropathy, hearing impairment, and mild biochemical abnormalities	Homozygous NM_003846.3:c.2T>G/p.Met1?	Segregation and likely pathogenic classification reported; functional evidence remains limited [46]

## Data Availability

No new data were created or analyzed in this study. Data sharing is not applicable to this article.
